# FASNet: Feature alignment-based method with digital pathology images in assisted diagnosis medical system

**DOI:** 10.1016/j.heliyon.2024.e40350

**Published:** 2024-11-13

**Authors:** Keke He, Jun Zhu, Limiao Li, Fangfang Gou, Jia Wu

**Affiliations:** a School of Computer Science and Engineering, Changsha University, Changsha, 410003, China; b Hunan University of Medicine General Hospital, Huaihua, 418000, China; c Collaborative Innovation Center for Medical Artificial Intelligence and Big Data Decision Making Assistance, Hunan University of Medicine, Huaihua, 418000, China; d State Key Laboratory of Public Big Data, College of Computer Science and Technology, Guizhou University, Guiyang, 550025, China; e Research Center for Artificial Intelligence, Monash University, Melbourne, Clayton, VIC, 3800, Australia

**Keywords:** Feature alignment, Digital pathology images, Assisted diagnosis, Deep learning, Insufficient annotation sets

## Abstract

Many important information in medical research and clinical diagnosis are obtained from medical images. Among them, digital pathology images can provide detailed tissue structure and cellular information, which has become the gold standard for clinical tumor diagnosis. With the development of neural networks, computer-aided diagnosis presents the identification results of various cell nuclei to doctors, which facilitates the identification of cancerous regions. However, deep learning models require a large amount of annotated data. Pathology images are expensive and difficult to obtain, and insufficient annotation data can easily lead to biased results. In addition, when current models are evaluated on an unknown target domain, there are errors in the predicted boundaries. Based on this, this study proposes a feature alignment-based detail recognition strategy for pathology image segmentation (FASNet). It consists of a preprocessing model and a segmentation network (UNW). The UNW network performs instance normalization and categorical whitening of feature images by inserting semantics-aware normalization and semantics-aware whitening modules into the encoder and decoder, which achieves the compactness of features of the same class and the separation of features of different classes. The FASNet method can identify the feature detail information more efficiently, and thus differentiate between different classes of tissues effectively. The experimental results show that the FASNet method has a Dice Similarity Coefficient (DSC) value of 0.844. It achieves good performance even when faced with test data that does not match the distribution of the training data. Code: https://github.com/zlf010928/FASNet.git.

## 1 Introduction

Semantic segmentation techniques have been widely used in medical image analysis in the field of medical-assisted diagnosis to assist doctors in early diagnosis and therapeutic intervention [[1], [2], [3]]. For example, tumor identification in orthopedics [4], tissue analysis in gynecological oncology [5], polyp segmentation in gastroenterology [6], and segmentation and analysis of skin lesions or tumors in dermatology [[7], [8], [9]]. Pathology images have been continuously researched in the field of image segmentation due to their ability to provide detailed tissue structure and cellular information, thus providing a more accurate basis for disease diagnosis.

However, the segmentation of digital pathology images is a challenging task [10]. This is mainly due to the complexity and variability of human tissue structure, which makes it difficult to accurately and finely segment cells, tissues, and structures in pathology images [11,12]. In addition, pathology slides may produce noise and artifacts during the production process, which can interfere with the physician's judgment [13]. The drawbacks of traditional recognition methods are particularly evident in regions and countries with scarce medical resources, mainly due to the following reasons.

1) Relying on pathology images for tumor diagnosis usually requires an experienced radiologist [14,15]. While many developing countries lack the human resources related to the diagnosis and treatment of osteosarcoma, it is difficult to spend significant manpower and time-consuming expenses in identifying and labeling osteosarcoma pathology images [16].
2) Due to the low-value density of pathology image data, the large number of redundant pathology images makes the physician's workload extremely heavy, with typically less than 20 images out of over 600 being able to provide diagnostic data for the condition [17], thus causing physicians to spend much time on images with deficient data information, and the cost of manually processing these pathology images is too high. The low-value density of pathology image data results in many redundant images, imposing a significant burden on physicians. Typically, fewer than 20 images out of over 600 can provide diagnostic data for the condition [18]. Consequently, physicians spend considerable time analyzing images with minimal information content, leading to high manual processing costs for pathology images.
3) Osteosarcomas come in a variety of locations, sizes, and shapes, with structures varying from patient to patient, and because osteosarcoma cells are occasionally indistinguishable from other normal human tissue cells [19]. Consequently, for precise tagging of osteosarcoma cell nuclei considerable time and effort are required by experienced physicians. Currently, hospitals lack the technology to efficiently divide the nuclei, and for inexperienced young doctors, misdiagnosis and misdiagnosis are inevitable, leading to delays in patient treatment.

To relieve physicians' work pressure, improve diagnostic efficiency compensate for the imbalance of medical resources, and to be able to reduce the possibility of wrong decisions made by less experienced physicians [20]. Efficient and accurate machine learning algorithms are urgently needed to enable the automatic segmentation of pathology images in artificial intelligence-assisted medical research. This will help reduce physician workload and shorten the time from patient visits to treatment completion [[21], [22], [23]]. Advances have been made in AI medical information assistance research with various deep neural networks, such as multiscale residual fusion networks to fuse feature maps of different resolutions [24], boundary-aware grid context-aware networks for enhanced texture feature extraction and tumor region localization [25], all of which have achieved great success in image segmentation of osteosarcoma.

Existing models suffer from overfitting on the dataset and poor generalization in the target domain dataset where the distribution is not visible, and the boundaries between classes are not obvious [26,27]. The accuracy of cell nucleus segmentation is insufficient. Increasing the dataset can improve the status quo. However, in practical medical diagnosis, it is often challenging to obtain adequate pathological images due to the expensive nature of pathological images and the difficulty in obtaining various styles of osteosarcoma cell shapes. This therefore leads to a lack of robustness and usefulness of the model. To address these issues, we used a domain generalization-based model aiming to obtain good generalization results using limited training samples [28].

Therefore, we propose a feature alignment-based detail recognition strategy (FASNet) for pathology image segmentation. It consists of a preprocessing model and a segmentation network (UNW). The preprocessing model enhances the image quality and reduces the degree of contamination of the picture through the noise estimation sub-module and the non-blind denoising sub-module. The UNW network inserts the feature center alignment and feature distribution alignment modules into the original encoder and decoder structure, which helps in the center-level and distribution-level alignment of the category features. This alignment is achieved through instance normalization and grouped categorization whitening of the feature map. Consequently, it promotes compactness among similar features and enhances separation between different classes of features. This, in turn, results in clearer boundaries between classes, improved recognition of feature detail information, and enhanced accuracy in cell nucleus segmentation—the capability of the program to produce excellent predictions with new data drawn from the true probability distribution.

The main contributions made in this essay are the following points.

1) The recognition model designed in this study is based on improved Feature Center Alignment (FCA) and Feature Distribution Alignment (FDA), which provide information about the target category, thus optimizing the accuracy of the model segmentation and its adaptability to new samples.
2) Through instance normalization and instance whitening, the module encourages compactness of features of the same class and separation between different classes of features, thus effectively achieving the elimination of different classes of styles, and the enhancement of feature recognition capabilities.
3) This study enhances finite feature recognition by eliminating global distribution differences and focusing on class-level semantic consistency, which results in clearer class boundaries and strong generalization ability in image segmentation tasks with different features.
4) Experiments using more than 4000 osteosarcoma pathology images showed that the method in this study has better performance. The predictive results of the model can provide informative support for the diagnosis of osteosarcoma in areas that lack medical resources.

## 2 Related work

Image segmentation has an extremely important role in medical assistive systems [29]. To enhance information transfer between layers, integrate feature maps, and combine multi-scale features for extracting complete feature information from images, Zhou et al. [30] introduced the U-Net++ network structure. This architecture improves feature map connectivity through the introduction of nested and dense jump connections. The UATransNet model [31] employs a multi-level guided self-aware attention module to eliminate irrelevant background information from images. Additionally, it enhances feature extraction by integrating a multi-scale jump connection into the model, alongside a convolution module that utilizes dense residual learning. BA-GCA [32] optimizes the segmentation capability of the model and localization of small areas of osteosarcoma in medical images by fusing multi-level features and focusing more on local details in the image. Recently, a vision transformer-based model has been developed for nuclei classification from histopathology images [33], and a different method using attention-aware adversarial networks [34] has been developed for nuclei segmentation.

Because the data annotation process is so costly, accurate and effective segmentation often relies on large amounts of finely annotated data, but some clinical diseases are rare in nature, so most of the current pathology image segmentation models are unsupervised learning, semi-supervised learning, and few-sample learning [35,36].

Kuang et al. [37] proposed a weakly supervised feature decomposition module based on semantic affinity to address the label symbiosis and positional adjacency problem in multi-class medical image segmentation. This approach enhances the performance of weakly supervised multi-class medical image segmentation using image-level labels. Chen et al. [38] introduced FDFUNet, a medical image segmentation network based on multiscale frequency domain filters. FDFUNet improves the perceptual field and depth of the network with a double residual depth separable convolution module (DRDAC), utilizes a multiscale frequency domain filter (MFDF) to extract multiscale information, and employs axial channel attention (ASCA) for channel weight computation. Wang et al. [35] proposed CRMEFNet, a novel network for medical image segmentation. CRMEFNet achieves high-precision segmentation of medical images through a coupled refinement module, multiscale exploration and fusion module, and cascaded progressive decoder.

Large digital pathology images typically suffer from poor domain adaptation and interpretability. Ming Y. Lu et al. [39] proposed a clustering-constrained attention multi-instance learning (CLAM) for learning from attention to accurately classify whole slides. The method can be used to localize well-known morphological features on WSI without the need for spatial labeling. Tingting Zheng et al. [40] proposed a weakly supervised Fast Medical Decision Making in Melanoma Histopathology Images (FastMDP-RL). The framework accelerates model inference by reducing the number of irrelevant patches identified in WSI. The results show that FastMDP-RL speeds up inference and accurately predicts WSI even in the absence of pixel-level annotations. Gabriele Campanelladengr et al. [41] on the other hand trained the system using reported diagnoses as labels, thus avoiding expensive and time-consuming pixel-by-pixel manual annotation. The test results show that the area under the curve is above 0.98 for a wide range of cancer types.

To alleviate the pressure of model generalization problems caused by data annotation uneven cell staining and the tightly packed and clumped morphological characteristics of cell nuclei, obtaining models with good generalization ability by the small amount of data is a difficult problem to solve for cell nuclei segmentation. Wu et al. proposed a double contrast learning network for few-sample learning [42], and constructed prototype-based and context-based dual contrast learning using auxiliary information from untargeted classes of medical images, enhancing the ability to learn image features and improving the utilization of few-sample data. Lou et al. [43] used a new consistency-based patch selection method to determine which pathology images are most beneficial for training. Using more training samples synthesized using GAN and a small number of pathology images requiring annotation, the existing nucleus segmentation model was trained using the enhanced samples described above, possessing the same performance as fully supervised. Chen et al. [44] introduced a novel framework named SIFA for unsupervised domain adaptation in medical image segmentation. This framework integrates image alignment and feature alignment for end-to-end training by sharing feature encoders through adversarial learning. By doing so, it effectively mitigates inter-domain variance and enhances segmentation performance in the target domain.

Few-shot learning shows unique advantages in data-limited modeling tasks. Hao Quan et al. [45] proposed a dual-channel prototype network (DCPN) for the effective classification of pathology images with limited data. DCPN combines a pyramidal vision transformer and convolutional neural network for extracting multi-scale pathology features, which improves the generalizability of prototype representations. With the significant advancements in neural networks, pathological image segmentation has found increased clinical utility in the precise diagnosis and prediction of osteosarcoma. This capability serves as a crucial aid in formulating subsequent treatment strategies for patients, thereby enhancing their survival rates. However, in medical image processing, gathering large annotated datasets of cases is frequently a daunting challenge. Additionally, staining and annotating pathological images is laborious and costly. Therefore, most models do not work well when faced with a variety of test data that are not consistent with the distribution of the training data.

To cope with the lack of datasets, inconsistency in the distribution of training data and test data faced in osteosarcoma pathology images in semantic segmentation, and to address the wide variety of data distributions that the model may face at the time of testing, we employ two class information modules for normalization and whitening. The model achieves central-level, distribution-level, and semantic-level feature alignment by augmenting and varying the data, which alleviates the semantic conflict problem caused by class feature mismatch and still helps the model maintain the evaluation indicators of the model in the training data as much as possible when facing various test data with inconsistent distribution with the training data.

## 3 System model design

**Fig. 1:**
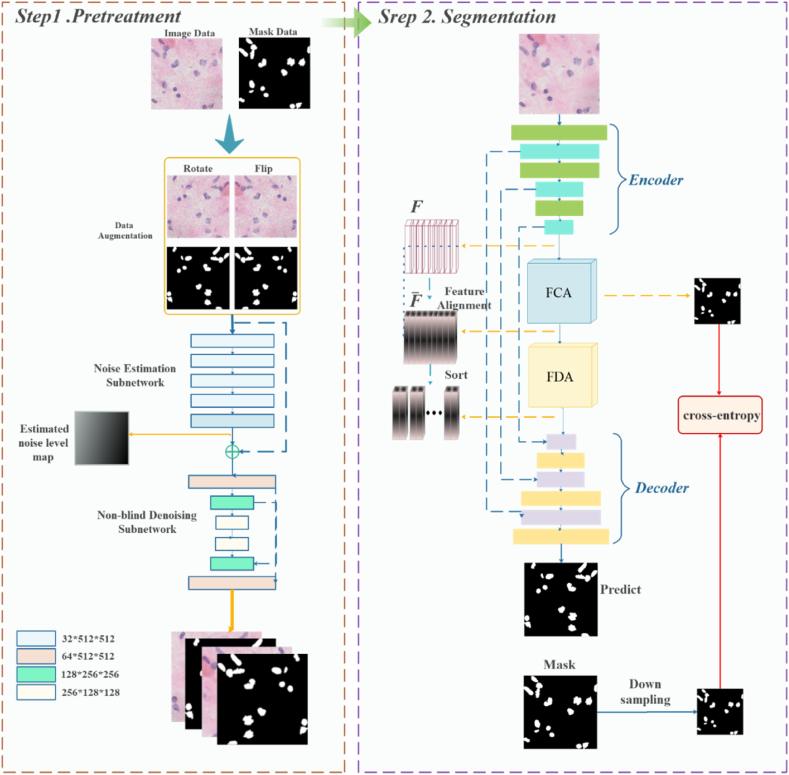
Pathology image segmentation framework.

In realistic medical pathology image recognition, it is impossible to know the data distribution status of the segmented pathology images, which means that model predictions are faced with multiple distribution types in the test data [46]. Osteosarcoma cells usually have distinct differences compared to normal cell nuclei. First, the nucleus of an osteosarcoma cell is usually larger than the nucleus of a normal cell and is irregularly shaped with non-smooth edges. Second, the chromatin in the nuclei of osteosarcoma cells is unevenly distributed, resulting in some areas showing dark staining and others showing light staining [47,48]. In addition, there may be differences in the number and size of nucleoli in the nuclei of osteosarcoma cells compared to normal cells. As a result, osteosarcoma pathology images have significant diversity, uneven staining, and complex background structure, and pathology images often have high cell density, cell overlap, and cell clumping, which exacerbate the difficulty of segmenting individual cell nuclei. The segmentation capability of the model varies significantly across different images. Existing methods fail to achieve clear class boundaries in cell nucleus segmentation, resulting in unclear decision boundaries and producing blurred or incorrect segmentation results. Therefore, we adopt the technique of feature alignment. As shown in Fig. 1, we initially perform data enhancement on the limited data by rotating and flipping the data. Additionally, we integrate FCA and FDA modules into the original image preprocessing, encoder, and decoder model architecture. We introduce undistorted image appearance by emphasizing the class information of feature images, conducting inter-class region normalization and group whitening guided by class information, performing feature center alignment, distribution alignment, and eliminating feature correlations. The model feature recognition capability and generalization capability are enhanced.

### 3.1 Data pre-processing

Noise inevitably occurs during image generation, transmission, and storage, affecting the image and leading to image degradation [49,50]. The presence of noise increases the difficulty of digital pathological image recognition and analysis. Therefore, we need to perform data pre-processing using an effective denoising technique, which should be appropriate for the images and employed with appropriate parameters [51], to reduce the adverse effects on the subsequent processing of medical images. Many DNN networks have been experimentally proven to have good results for image noise reduction, to decrease noise and enhance the robustness of the UNW, we used the CBDNet model for pathology image denoising [52].

**Fig. 2:**
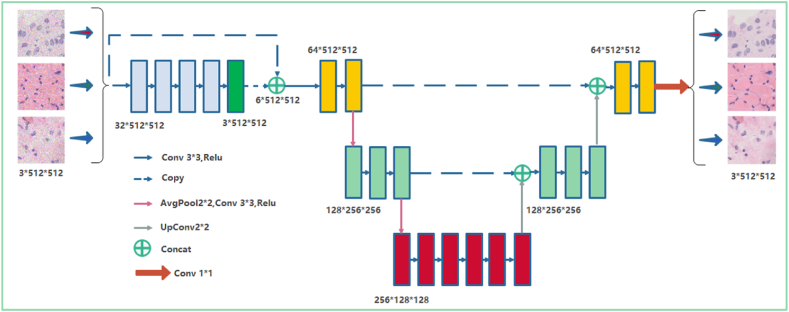
Image pre-processing model.

The model is composed of two parts, the first part for noise estimation and the second part for non-blind denoising. As shown in Fig. 2, the network architecture consists of a fully convolutional network (FCN), i.e., a noise estimation subnetwork, and a U-Net, i.e., a non-blind denoising subnetwork. First, FCN converts the input image y into an estimated noise image $\bar{y}$. Then, the y and $\bar{y}$ are combined as the input of the non-blind denoising sub-network, and the denoised high-quality image is obtained by the convolutional network afterward.

Enhance the model by incorporating both real noise images and synthetic noise images as input data during training. The CBDNet model integrates real-world noise distribution learning with synthetic noise image utilization to mitigate the scarcity of noisy image data. Through the CBDNet model training, effective denoising procedures can be applied to osteosarcoma pathology images, enhancing damaged image quality and preventing noise-induced distortion in the image segmentation structure, thereby minimizing segmentation boundary errors that may impact patient diagnosis and treatment outcomes adversely. Extending the CBDNet model training process involves alternating the use of two types of noisy images as input data, thereby expanding the training dataset. This enables the model to learn features from both real and simulated noise, thus improving its feature extraction capability and generalization to various noisy images encountered in real-world scenarios. Preprocessing osteosarcoma pathology images can enhance image quality, diminish image contamination, and furnish the image segmentation model with realistic features and details.

### 3.2 Data augmentation

It is a common technique to expand data through data augmentation to enhance the model generalization capability [[53], [54], [55]]. Data augmentation is generally done by having two common techniques [53]. The first one expands the data set by transforming the training data in various forms to achieve a diverse data distribution, such as panning, rotating, mirror flipping, adding noise, etc. to the sample images. Expanding the data by geometrically changing the data can provide a certain degree of generalization of the model to test sets with unknown data distribution. However, improving the adaptability of the algorithm to fresh samples by enhancing the training data with this method is inherently flawed. The technique is not able to cover all the different scenarios that may be present in the target domain and cannot obtain samples of osteosarcoma pathology images for all distributions of data, thus leading to deficiencies in the generalization performance of the model in unknown data. Another current approach is to utilize a combination of normalization and whitening of images to enhance model generalization by normalizing the feature distribution of the feature map to achieve data augmentation [28]. Instance Normalization (IN) and Instance Whitening (IW) are common approaches to adjusting the feature distribution. The correlation between features can be eliminated by IN and IW so that the model can generalize well even when the texture and color of the image change. In this paper, we enhance the model generalization by improving the IN and IW methods for data augmentation and centralizing and distributing the feature distribution.

Instance Normalization (IN) is a simple algorithm for adjusting feature distribution, acting on a single image by computing the mean and standard deviation of all pixels of a single image. There are various IN methods applied to different medical images [[56], [57], [58]]. Since IN does not mix batch and inter-channel features, it is particularly well-suited for adjusting the feature distribution of individual pathology images. This ensures that each sample image possesses its unique feature distribution, thereby enhancing feature information. IN normalizes the features of each channel of a single image to mitigate feature mismatches due to style changes, and IN can filter complex appearance patterns, introducing appearance invariance [59]. Equation (1) can represent the specific process of IN:

(1) $$\left\{ \begin{array}{c} \text{IN}\left(x_{\text{tijk}}\right)=\frac{x_{\text{tijk}}-\mu_{\text{ti}}}{\sqrt{\Sigma_{\text{ti}}^{2}+\beta }}\\ \mu_{\text{ti}}=\frac{1}{HW}\sum_{l=1}^{H}\sum_{m=1}^{W}x_{\text{tilm}}\\ \Sigma_{\text{ti}}^{2}=\frac{1}{HW}\sum_{l=1}^{H}\sum_{m=1}^{W}{\left(x_{\text{tilm}}-\mu_{\text{ti}}\right)}^{2} \end{array}\right.$$

$X_{tijk}$ represents the feature map on the i-th channel feature values of the t-th sample at the spatial location (j, k). Using IN only achieves the central alignment of the feature distribution, but not the joint distribution of the features. IN only obtains the alignment at the central level ignoring the joint distribution between different channels. Instance normalization only normalizes the features and does not take into account the correlation between channels. This means that applying instance normalization to the network may be insufficient for domain generalization, as no feature covariance is given consideration [60].

Instance Whitening (IW) can eliminate the linear correlation between the features of each channel so that well-clustered features with uniform distribution can be formed after using IW. The specific process of IW can be expressed by the following equation (2).

(2) $$\left\{ \begin{array}{c} L_{IW}=\sum_{n=1}^{N}\|\Sigma -Ι|{|}_{1}\\ \Sigma =\left(\begin{array}{ccc} \sigma \left(M_{n,1},M_{n,1}\right) & \ldots & \sigma \left(M_{n,1},M_{n,c}\right)\\ \vdots & \ddots & \vdots \\ \sigma \left(M_{n,c},M_{n,1}\right) & \cdots & \sigma \left(M_{n,c},M_{n,c}\right) \end{array}\right)\\ \sigma \left(M_{n,j},M_{n,k}\right)=\frac{1}{HW}\sum_{h=1}^{H}\sum_{w=1}^{W}\left(M_{n,j,h,w}-\mu_{n,j}\right)\left(M_{n,k,h,w}-\mu_{n,k}\right) \end{array}\right.$$

**Fig. 3:**
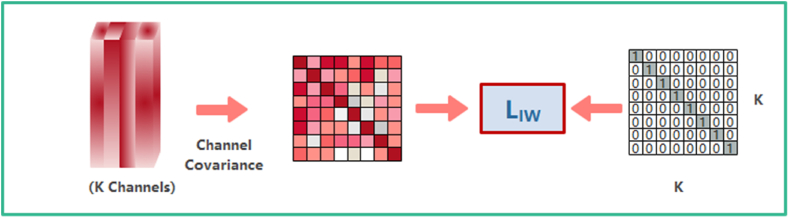
The effect of IW.

Where Σ represents the covariance between the individual channels of the n-th sample in a batch, is essentially a representation of the correlation between the individual channels on the feature map. IW eliminates the correlation between channels by equation (2). The effect of IW is shown in Fig. 3.

Since adjacent pixels in an image are strongly correlated with each other, the whitening transform removes the correlation between features and makes each feature have unit variance. Feature whitening effectively eliminates the change of style information in images due to panning, style-shifting, etc., which can boost the model's performance in extracting feature details. Nevertheless, it is not simple to adopt the whitening transform directly to enhance the robustness of the network, since the whitening operation on the feature images does not differentiate the feature information, which may consequently result in the elimination of all feature information [32].

However, after combining IN and IW, the joint feature edge distributions from different domains can be aligned. Although the feature edge distributions are aligned, the conditional distributions remain unaligned, and the distributions of each category are still mixed to the extent that they are difficult to distinguish.

The global alignment strategy that combines IN and IW lacks consideration for the consistency of local feature distributions. Features belonging to different categories, which were initially well separated in the original model architecture, may become mapped together after IN and IW processing. This could result in category confusion, impacting the model's feature recognition ability, especially when applied to a specific target image. This semantic inconsistency inevitably leads to performance degradation on the invisible target domain, and the performance is not satisfactory in the segmentation of osteosarcoma pathology images.

Therefore, the model used in this paper is both center-aligned and distribution-aligned, and the category information is introduced based on IN and IW so that the distribution of each category can be well distinguished in the feature space and the graph is not distorted.

### 3.3 Cell nucleus segmentation network

#### 3.3.1 FCA

**Fig. 4:**
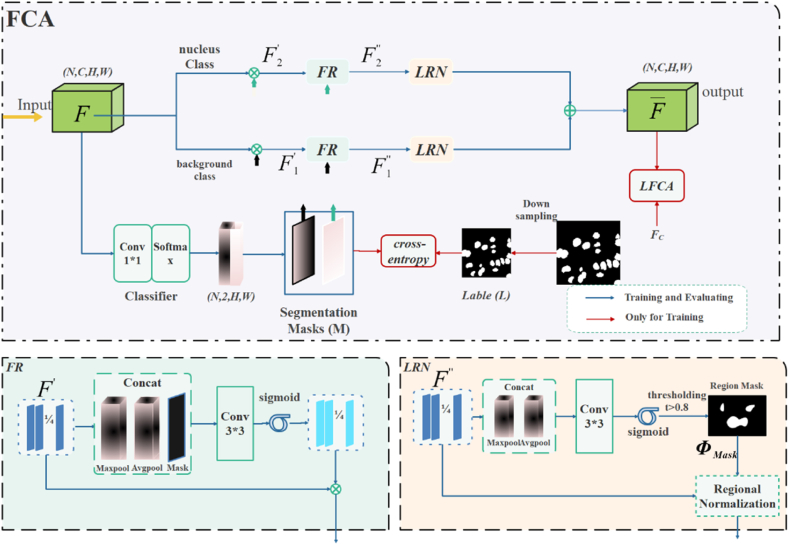
The FCA module structure, FR, and LRN module structure.

FCA converts the feature map into a category-level center-aligned feature map, as shown in Fig. 4. It is done by normalizing each category for each channel of the feature map for each sample.

As shown in Fig. 4, $F_{C}$ is obtained by normalizing the encoder-extracted feature map F at the category level by instances, as shown in Eq (3).

(3) $$\left\{ \begin{array}{c} F_{C}=\frac{F_{n,m}^{c}-\mu_{n,m}^{c}}{\sqrt{\Sigma_{n,m}^{c}+\beta }}*\alpha^{c}+\varepsilon^{c}\\ \mu_{n,m}^{c}=\frac{1}{\left|L\left(c\right)\right|}\sum_{L\left(c\right)}F_{n,m}^{c}\\ \Sigma_{n,m}^{c}=\frac{1}{\left|L\left(c\right)\right|}\sum_{L\left(c\right)}{\left(F_{n,m}^{c}-\mu_{n,m}^{c}\right)}^{2} \end{array}\right.$$

Where $F_{n,m}^{c}$ denotes the set of feature values of the spatial location to which the c-th class on the feature map $F_{n,m}^{c}$ belongs. α and ε denote the instance normalized scaling and translation weights, respectively. $\mu_{n,m}^{c}$ and $\Sigma_{n,m}^{c}$ represent the mean and standard deviation obtained from the calculation of the m-th channel in the n-th sample, and the c-th class of label. The feature map F and $M_{C}$ do Hadamard Product to highlight the category region to get C different feature maps $F_{C}$.

The Feature Refinement (FR) module is introduced to make category-level feature refinement. Fig. 4 illustrates the specific operation of FR, which takes the feature map Fc of the c-th category, and pools it by maximum pooling and average pooling, respectively. The pooled result and its corresponding $M_{C}$ are concatenated along the channel axis. Subsequently, a 3∗3 convolution is applied, followed by activation through the sigmoid function. The resulting convolution is then summed with the original $F_{C}$ to obtain the refined feature map.

To further refine the category-level center alignment, a Learnable Region Normalization (LRN) module is added to normalize the category feature regions. LRN splices the $F_{C}$ after maximum pooling and average pooling, and then obtains the Region Mask after sigmoid activation and thresholding, and later region normalization is performed. The operation is repeated for each class of branches and finally the results of each branch are summed up.

$F_{C}$ can obtain category-level normalization because of the availability of truth-valued labels Y. Since labels are difficult to obtain in the testing phase, the FCA model adds a pre-segmented branch to introduce the correct semantic category information. The feature map F extracted by the encoder will be multiplied with the channels corresponding to the categories of the pre-segmented branches respectively to emphasize the features in the regions of the feature map corresponding to the semantic categories. However, the feature maps after direct multiplication may incorrectly emphasize some features that do not belong to the current category, therefore, this paper proposes a category-level feature optimization module (FR) to improve the feature maps of pathology images. Maxpool and Avgpool in FR are extended in the channel dimension to smooth the error information introduced by pre-segmentation to some extent. The FR-optimized features are sent to the region normalization (LRN) for the final normalization operation [61]. And, the feature map is subjected to two pooling operations to extract valid feature descriptions. Subsequently, thresholding is performed using a threshold with parameter t=0.8. The resulting selected region is called the region mask, which is essentially predicted by the module Y(c) and represents the nuclei and background regions in the pathology image. The normalization result is shown in equation (4):

(4) $$\bar{F}=\sum_{c=1}^{C}\text{LRN}\left(F_{c}^{\prime \prime },\Phi_{mask}\right)$$

**Fig. 5:**
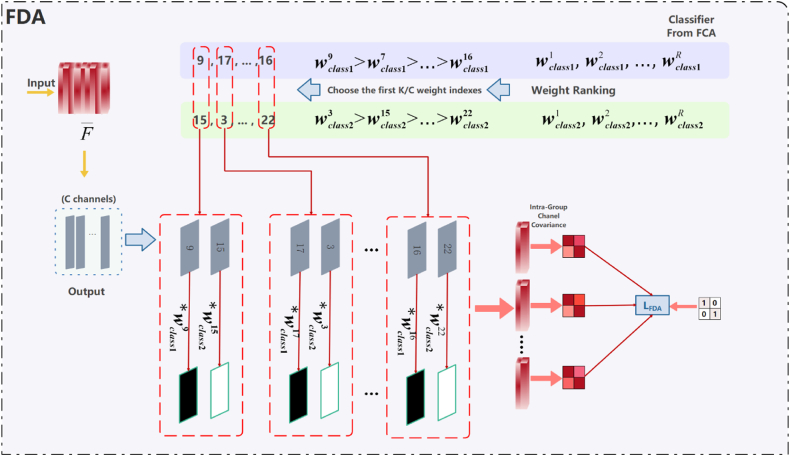
FDA module structure.

Among them, LRN corresponds to the features in the mask area of branch c, such as normalization, while the remaining spatial features remain unchanged. The overall objective function of FCA is equation (5).

(5) $$L_{F\text{CA}}=CE\left(M,L\right)+\|\bar{F}-F_{C}|{|}_{1}$$

where CE is cross-entropy in Fig. 5, and the second term serves to make the normalization result of FCA approximate $F_{C}$. without the truth labels, so that the pathology images in the test set are normalized as close as possible to the labeled data when the category-level instances are normalized.

#### 3.3.2 FDA

To further enhance the de-correlation between channels, features that are already semantically centered are further aligned distributively. The FDA module is added to perform the re-alignment of channels, ensuring distributed alignment with subtle changes in semantic content for each group containing channels associated with different categories [28].

FDA is improved based on IW, which is mainly grouped by the information of semantic categories, as shown in Fig. 5. The semantic information comes from the weight of the categories in the $F_{C}$, which represents the significance of each channel in different categories of feature maps, that is, it indicates the type of information of the channel. The grouped features can be obtained by sorting this, and then taking out the corresponding feature maps in turn. Channels from different categories are grouped together, and the correlation between these channels is removed to enhance the representational capacity of each individual channel. Categories are aligned in a distributed manner. A total of K/2 groups, each containing 2 channels, were selected. After grouping, it is sufficient to calculate the loss function $L_{FDA}$ that is consistent with the form of $L_{IW}$ in Fig. 3.

FDA aims to achieve further semantic distribution-level alignment for class-centered aligned features, which will process the normalized features in the FCA module to align the full local marginal distribution with the conditional distribution. FDA delves into the correlation between channels and classes to differentiate between class attributes to reduce the correlation that exists between the nucleus and the background and to enhance the segmentation ability of the model in the unknown target domain.

Specifically, the FDA finds channels with high relevance to each category according to the weights of the model classifier. These highly correlated category channels are assembled into a grouping, denoted by ρ. ρ(c, i) represents the i-th highly correlated channel index associated with the c-th category. As shown in formula (6):

(6) $$\left\{ \begin{array}{l} P_{n}^{m}=\left[{\bar{F}}_{n,\rho \left(1,m\right)}*w_{1}^{\rho \left(1,m\right)};{\bar{F}}_{n,\rho \left(2,m\right)}*w_{2}^{\rho \left(2,m\right)};\cdots ;{\bar{F}}_{n,\rho \left(C,m\right)}*w_{C}^{\rho \left(C,m\right)}\right]\\ L_{\text{FDA}}=\frac{1}{N}\sum_{n=1}^{N}\sum_{m=1}^{\frac{K}{C}}{\left\|\Sigma \left(P_{n}^{m}\right)-I\right\|}_{1} \end{array}\right.$$

Where $P_{n}^{m}$ is the m-th feature grouping of the nth sample in the FDA module and w is the corresponding classifier weight. The distribution-level alignment of semantic features is achieved by erasing the correlation of different class channels within the group, while protecting the feature structure information.

#### 3.3.3 UNW model architecture

**Fig. 6:**
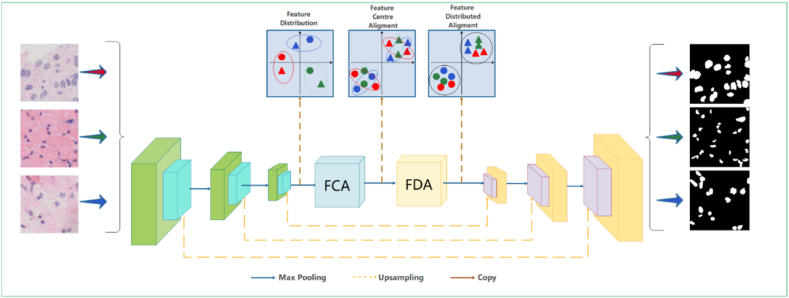
Image segmentation system framework (UNW).

Fig. 6 illustrates the insertion of two plug-and-play modules, FCA and FDA, into the original model's encoder and decoder structural framework. This modification addresses the model's poor generalization ability caused by distribution differences between the training set and the unknown target segmentation dataset. This approach can enhance the feature extraction capability of the original model, improve the algorithm's adaptability to new samples, improve inaccuracies in cell nucleus segmentation in pathological images with unknown osteosarcoma distributions, and ultimately enhance the accuracy of cell nucleus segmentation.

The UNW network has a strong practicality with the realistic premise that we cannot know any information about the target domain including the data of osteosarcoma pathology images, and collecting data covering all possible scenes in the target domain is both expensive and difficult, and even impossible to accomplish in medical images [62,63]. Therefore, the model is more likely to be overfitted in fewer training sets, and the model probably faces a variety of data distribution images when actually segmenting pathological images. If the model is not capable of extracting enough information about category features, its accuracy of obtaining target images after segmenting cell nuclei will be drastically reduced, and it will not be able to accomplish the task of assisting doctors in diagnosis. Existing approaches extensively eliminate global distribution differences, but do not take into account semantic consistency at the category level, resulting in limited feature recognition capability of the model. The encoder-decoder structure is adopted to embed the FCA and FDA modules. FCA promotes compactness within categories through feature center alignment, while FDA achieves separation between different categories through distributed alignment of features, resulting in effective style elimination and robust feature recognition. This enhancement greatly facilitates the task of cell nucleus segmentation in osteosarcoma pathology images.

UNW reduces the disparity in probability distribution between the training data and the pathological images of real osteosarcoma patients through feature alignment. This process aims to simulate the real-world data distribution as much as possible with the limited training data.

## 4 Experimental results

### 4.1 Experiment description

In our experiments, we utilized over 4000 histopathology images of osteosarcoma from the Research Center for Artificial Intelligence, Monash University. Initially, there were a total of 1006 digital pathology images, originating from 23 patients, with 19 diagnosed with osteosarcoma and the remaining 4 with other conditions. Among the osteosarcoma cases, there were four stages: 1 case in stage I, 4 cases in stage II, 6 cases in stage III, and 8 cases in stage IV. We localized the regions of interest in the digital case images and performed random cropping. Subsequently, the cropped images were enlarged to obtain standard format images of size 512∗512. Initially, we obtained 3527 images, out of which 2877 were deemed valid after manual screening, and were annotated by 7 pathology experts to create the dataset labels. Due to the risk of overfitting in deep learning models caused by insufficient datasets, we augmented the dataset by randomly flipping images horizontally and vertically, and rotating them by 90, 180, and 270°. The augmented dataset comprised a total of 4328 images, which were divided into training and testing sets in a 7:3 ratio.

To validate the model's generalization capability, we also conducted experiments on the TNBC dataset [64]. The TNBC dataset is an open dataset containing annotations of various cell types, including normal epithelial and myoepithelial breast cells, infiltrating cancer cells, fibroblasts, endothelial cells, adipocytes, macrophages, and inflammatory cells. The dataset consists of 50 histopathology images, with a total of 4022 annotated cells. The maximum number of cells in a single sample is 293, while the minimum is 5, with an average of 80 cells per sample and a relatively high standard deviation of 58. The dataset was also divided into training and testing sets in a 7:3 ratio. Our network performs a total of 200 iterations with 512∗512 input images and a batch size of 6.

Table 1 displays the specific operating environment and parameters of the experiment.

**Table 1:** Implementation details.

	Aspect	Statistic
Model Parameter	optimizer	SGD
	learning rate	5e-4
	momentum	0.9
	weight decay	5e-4
	class	2
Operating Environment	Operating System	Ubuntu20.04 LTS
	GPU	NVIDIA RTX 3090
	Memory	50G

### 4.2 Evaluation metrics

In model training and testing, we quantified the results of model segmentation of cell nuclei. As shown in Table 2, we utilize the confusion matrix to accurately evaluate the results of cell nuclei segmentation of pathological images. Utilizing the four datasets provided by the confusion matrix, we compute the accuracy, precision, Recall, IOU, and DSC metrics of the model. Furthermore, we analyze the ratio of segmented nuclei and background pixels to all pixels in the image, as well as the similarity between predicted and actual labels. This analysis allows us to further investigate the model's effectiveness and improvement effects discussed in the paper [65,66]. The number of parameters and floating-point operations (FLOPs) of the model were also calculated to understand the environment required for the model operation and to analyze the practical application of the model [67].

**Table 2:** Confusion matrix.

	Actual: YES	Actual: NO
Predicted: YES	TP	FP
Predicted: NO	FN	TN

Acc is the proportion of all samples that are correctly categorized. It is defined as equation (7):

(7) $$Acc=\frac{TP+TN}{TP+TN+FP+FN}$$

Pre denotes the ratio of accurately segmented nuclei and background pixels to all pixels in the image, defined as, defined as equation (8):

(8) $$Pre=\frac{TP}{TP+FP}$$

Recall as represents the percentage of the total number of kernel pixels in the labeled images that are correctly segmented by the proposed method, also known as sensitivity, is defined as equation (9):

(9) $$Recall=\frac{TP}{TP+FN}$$

IOU is the similarity between the predicted nucleus region and the real nucleus region. We introduce $I_{1}$ as the real region and $I_{2}$ as the predicted region, defined as equation (10):

(10) $$IOU=\frac{I_{1}\cap I_{2}}{I_{1}\cup I_{2}}$$

DSC is an ensemble similarity measure, which is usually used to calculate the similarity of two samples, and the calculation method is defined as equation (11):

(11) $$DSC=2*\frac{\left|I_{1}\cap I_{2}\right|}{\left|I_{1}\left|+\right|I_{2}\right|}$$

### 4.3 Image pre-processing

**Fig. 7:**
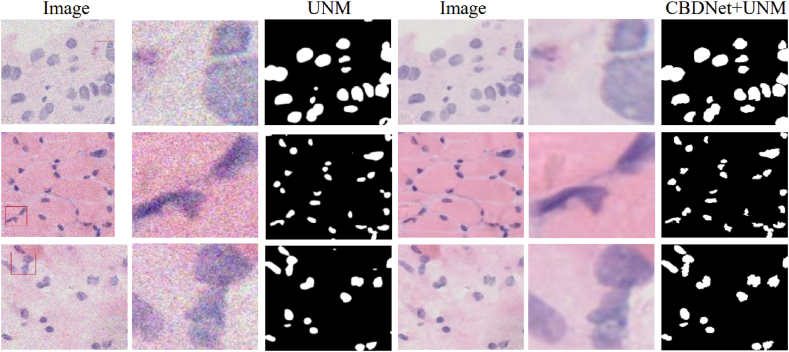
Comparison of segmentation effect with and without pre-processing.

As shown in Fig. 7, the original pathology image contains noise, which interferes with detail extraction during feature extraction by the model. This interference leads to segmentation errors at cell nucleus boundaries, thereby impacting segmentation accuracy. After denoising by the preprocessing model, we observe that the pathology images are segmented, resulting in target images that closely resemble the images manually labeled by experts. Furthermore, the edge processing and shape of the cell nuclei in the osteosarcoma pathology images are more accurate. After image pre-processing, denoising the pathology images can enhance the image quality, provide more accurate feature information for the segmentation model, and improve the accuracy of the model in segmenting cell nuclei. The evaluation index of whether the images were preprocessed or not from Table 3 can better quantitatively evaluate the effect of preprocessing. By comparison, it was found that the model can improve the accuracy rate of 0.6 % and the precision rate of 4 %, indicating that the CBDNet model preprocessing of pathological images can optimize the segmentation capability of the model and enhance the accuracy of cell nuclei segmentation, which can provide more effective assistance for patients in treating osteosarcoma.

**Table 3:** Evaluation indicators.

Methods	Acc	Pre	DSC	Recall	IOU
UNW	0.966	0.816	0.776	0.772	0.638
CBDNet [52] +UNW	0.972	0.856	0.844	0.880	0.704

### 4.4 Splitting effect

**Fig. 8:**
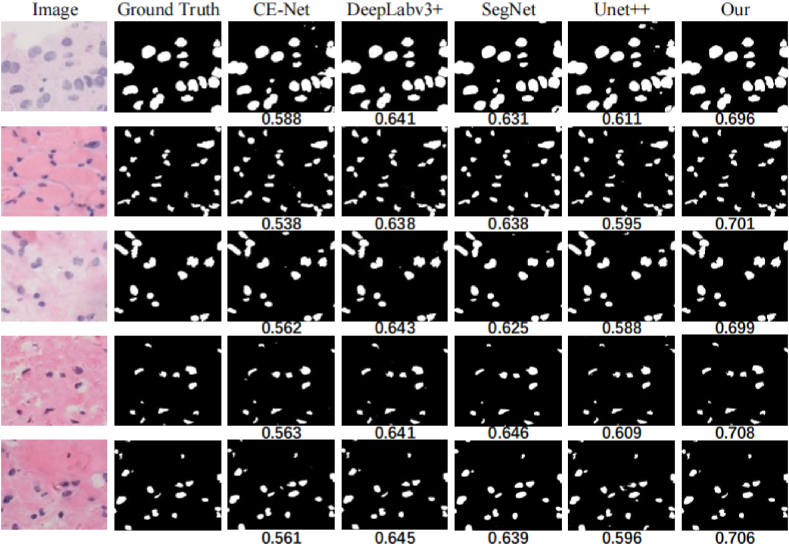
Different models for segmenting cell nuclei images.

The models were trained on the same training set and then applied to the test set images to segment the cell nuclei and assess the model's performance. The first column in Fig. 8 shows the denoised original image, the second column shows the true labels and the third to seventh columns show the predicted masks. As shown in Fig. 8, the true labels were annotated by experienced doctors. The segmentation results suggest that images segmented by the FASNet model exhibit maximum similarity to the true labels, and the model accurately segments nuclei regardless of their position or shape in the image. Even when the shape of the cells is complex. The model also performed accurate segmentation, and the segmented images are almost indistinguishable from the real labels, and the results show that FASNet can be helpful for medical diagnosis. A comparison of the IOU parameters at the bottom of the image reveals that FASNet has an obvious advantage in pathological image segmentation, indicating that the segmented image obtained by the model is most similar to the true label.

As shown in Table 4, we have utilized some general evaluation criteria to analyze and evaluate the performance of the algorithm for the purpose of accurately measuring the effectiveness of the model. To reduce randomness and bias and enhance the stability and reliability of model performance evaluation, we employed five-fold cross-validation to assess the models. We adopt accuracy, precision, recall, F1 score, IOU and DSC as evaluation metrics to assess the segmentation capability of the model and used Params and FLOPs to measure the parameters of the network. To quantitatively compare the performance of the models, Table 4 shows the computational results of each algorithm in different datasets. The "±" data are the standard deviation of the metric. FASNet performs well on each evaluation metric. From the table, it is evident that our FASNet outperforms other models in terms of Acc, precision, and IOU metrics. On our dataset, FASNet achieves an IOU of 0.704 and an Acc of 0.972. Additionally, FASNet exhibits lower standard deviation compared to other models, indicating greater stability in segmentation. Although CE-Net achieves a DSC of 0.889, its IOU is notably low at only 0.562. The RefineNet network achieves the highest recall value of 0.887, yet its relatively large standard deviation suggests insufficient confidence in its predictions. The evaluation data show that FASNet has good application in segmenting the nucleus region can provide guidance to physicians.

**Table 4:** Comparison of different algorithms.

Method	Accuracy		Precision		DSC		Recall		IOU	
	Our	TNBC	Our	TNBC	Our	TNBC	Our	TNBC	Our	TNBC
**Attention-Unet** [68]	0.963±	0.951±	0.708±	0.687±	0.84±	0.728±	0.732±	0.767±	0.588±	0.578±
	0.09	0.08	0.06	0.06	0.06	0.08	0.06	0.08	0.08	0.09
**CE-Net** [69]	0.945±	0.921±	0.641±	0.536±	**0.889±**	0.732±	0.747±	0.776±	0.562±	0.585±
	0.04	0.06	0.05	0.08	**0.05**	0.06	0.04	0.07	0.04	0.06
**DeepLabv3+** [70]	0.969±	0.948±	0.73±	0.69±	0.777±	0.765±	0.872±	0.876±	0.642±	0.614±
	0.05	0.04	0.05	0.05	0.06	0.04	0.08	0.07	0.05	0.06
**RefineNet** [**71****]**	0.971±	0.964±	0.724±	0.689±	0.784±	0.786±	**0.887±**	**0.879±**	0.655±	0.64±
	0.05	0.06	0.06	0.06	0.05	0.07	**0.07**	**0.09**	0.06	0.04
**SegNet** [72]	0.965±	0.957±	0.768±	0.721±	0.773±	0.761±	0.821±	0.801±	0.638±	0.626±
	0.04	0.08	0.06	0.06	0.09	0.08	0.07	0.05	0.07	0.09
**Unet++** [20]	0.961±	0.954±	0.681±	0.643±	0.735±	0.723±	0.87±	0.843±	0.596±	0.604±
	0.04	0.07	0.04	0.06	0.08	0.04	0.04	0.09	0.06	0.08
**TSCA-ViT** [73]	0.957±	0.953±	0.768±	0.74±	0.843±	0.852±	0.879±	0.864±	0.569±	0.593±
	0.08	0.09	0.06	0.06	0.08	0.07	0.06	0,08	0.09	0.07
**Our**	**0.972±**	**0.971±**	**0.856±**	**0.791±**	0.844±	**0.876±**	0.88±	0.873±	**0.704±**	**0.745±**
	**0.03**	**0.04**	**0.03**	**0.04**	0.05	**0.07**	0.04	0.05	**0.07**	**0.06**

**Fig. 9:**
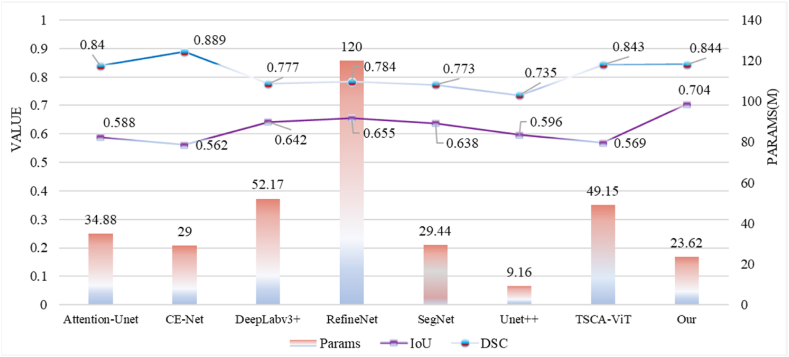
Comparison of IOU, DSC, and parameters of different models.

To more accurately analyze the segmentation performance of each model, we obtained line charts of IOU, DSC, and Params for each segmentation model through experiments, as well as comparison charts of Recall and FLOPs. From Fig. 9, it can be observed that our FASNet performs the best in terms of the IOU metric, indicating high segmentation accuracy of our model. Additionally, CE-Net exhibits better DSC than our model, but significantly lower IOU. We hypothesize that CE-Net may excel at predicting internal segmentation areas but lacks accuracy in segmenting boundaries, leading to a higher DSC but lower IOU. Furthermore, due to the imbalance in pathological data, with the background area being larger than the cell nucleus area, CE-Net may tend to predict larger segmentation areas, contributing to this phenomenon.

**Fig. 10:**
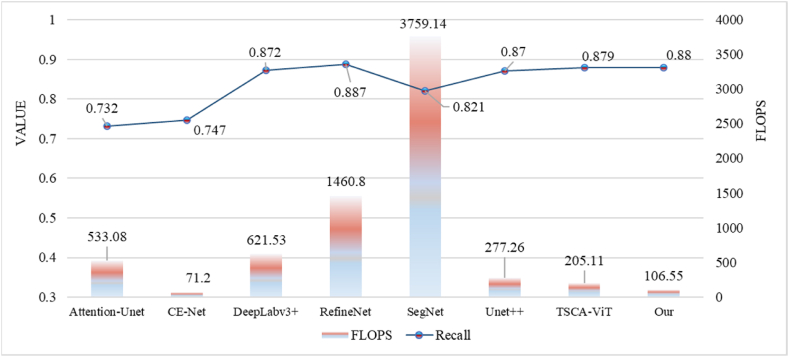
Comparison of Recall and FLOPs of different models.

In Fig. 10, the recall rate of FASNet performs well compared to other models, being only 0.007 lower than RefineNet, reflecting the high segmentation accuracy of our adopted model. While ensuring segmentation accuracy, FASNet has relatively fewer Params and FLOPs, with only 23.62 million parameters and 106.55 billion FLOPs, achieving lightweight model while maintaining accuracy. This significantly reduces training difficulty and application costs, minimizes model storage space and computational resources, and improves model efficiency and deployment flexibility. This enables our cell segmentation model to run smoothly on low-cost hardware.

**Fig. 11:**
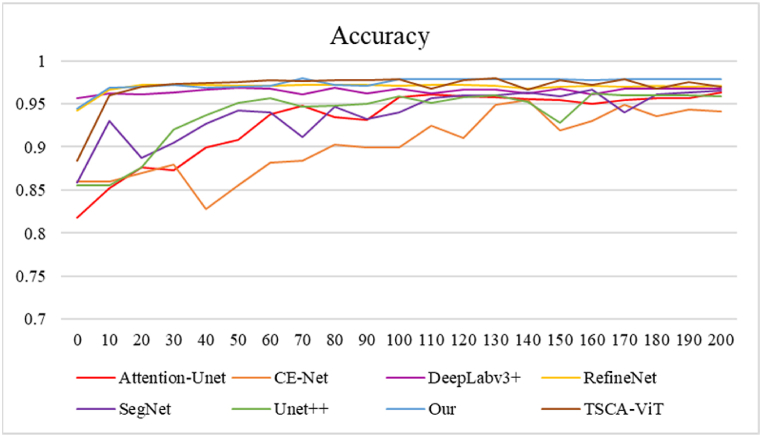
Accuracy of different models.

**Fig. 12:**
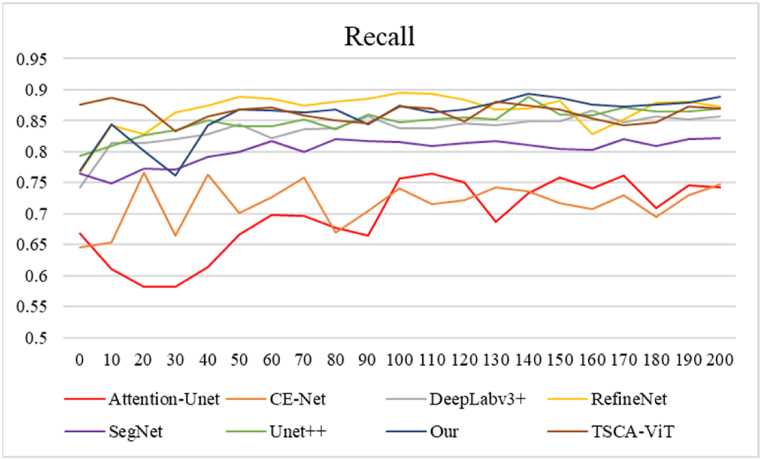
Comparison of Recall for different models.

Fig. 11, Fig. 12 show the advantage of FASNet in the iterations. Fig. 11 illustrates the accuracy of the different methods during the first 200 rounds of training. The image shows that CE-Net exhibits less stability and ultimately achieves the lowest accuracy. The FASNet approach is only marginally inferior to the DeepLabv3+ model in the initial training phase. However, after the 10th iteration, the accuracy values of FASNet quickly converge and surpass those of the other models. The accuracy remains relatively stable, reaching a final accuracy of 0.972. This indicates good results in cell nucleus segmentation in osteosarcoma pathology images, surpassing the segmentation performance of the other models. Fig. 12 shows the Recall of these models in the test set after 200 training sessions to stabilize the data. The images show that the values of FASNet in Recall perform better than the Recall of most of the models. Recall shows good results in segmenting cell nuclei in osteosarcoma pathology images, with clear boundaries between classes, and the model can find more target objects in the images, which has an advantage over the segmentation results of the rest of the models.

**Fig. 13:**
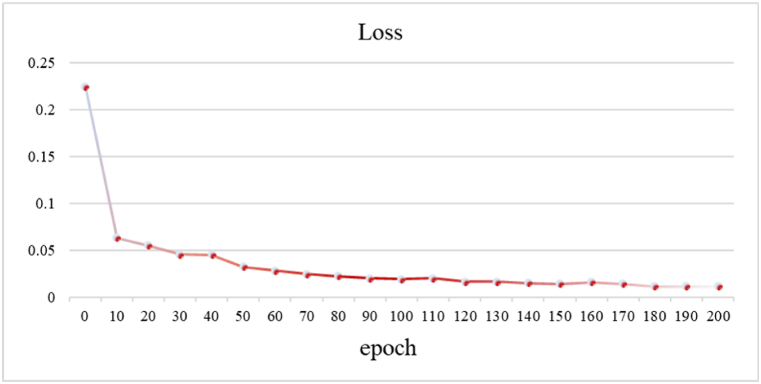
Trend of training loss over the epochs.

In Fig. 13, we have documented the loss values of the model at each epoch. As the training progresses, the loss gradually decreases, indicating the model's convergence during the learning process. Specifically, the initial loss value is 0.2247, which decreases to 0.0192 after 100 epochs, and further decreases to 0.0113 after 200 epochs. These results demonstrate the stability and convergence of our model during the training process.

### 4.5 Ablation experiments

**Fig. 14:**
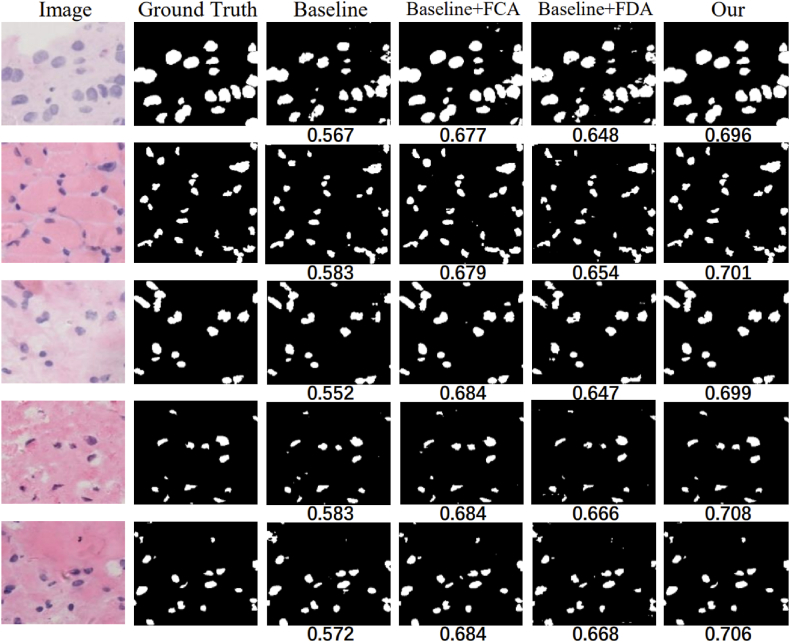
Effect of cell nucleus segmentation with different module combinations.

We conducted ablation experiments to better validate that the model was impacted by the inserted FCA and FDA modules. We investigated the individual contributions of the FCA and FDA modules to the overall performance. In our experiments, each of them contributes to a substantial improvement in generalization performance. Specifically, we observed that the accuracy, precision, DSC, Recall, and IOU of the model had significant improvements when using the FCA module and the FDA module alone and that the simultaneous use of FCA and FDA had the largest improvement in the segmentation efficiency of the model. From the data in Table 5, we can conclude that inserting both FCA and FDA can maximize the performance of the original model and can have a significant improvement on the segmentation results of pathological images. The graph of cell nucleus segmentation results in Fig. 14 demonstrates that the model consisting of the combination of modules we used has the best segmentation results. Ablation experiments show that adding FCA and FDA to the middle of the encoder and decoder helps to improve the adaptability of the original model to fresh samples. We are able to quantify the segmentation effect of the model also by considering the IOU parameters at the bottom of the image, and we can observe that the model with both FCA and FDA modules has the most powerful segmentation ability. Additionally, we conducted statistical hypothesis testing on the data, using the DSC score as an example. The original hypothesis was “the test set and training set have the same distribution”. A significance level of 0.05 was chosen. The final test yielded a p-value of 0.18, significantly higher than 0.05. Thus, we cannot reject the null hypothesis and accept the original hypothesis that “the test set and training set have the same distribution".

**Table 5:** Ablation experiments of FASNet.

Methods	Accuracy	Precision	DSC	Recall	IOU
Baseline	0.923	0.724	0.732	0.712	0.578
Baseline + FCA	0.955	0.809	0.808	0.813	0.689
Baseline + FDA	0.941	0.780	0.778	0.784	0.665
All	0.972	0.856	0.844	0.880	0.704

**Fig. 15:**
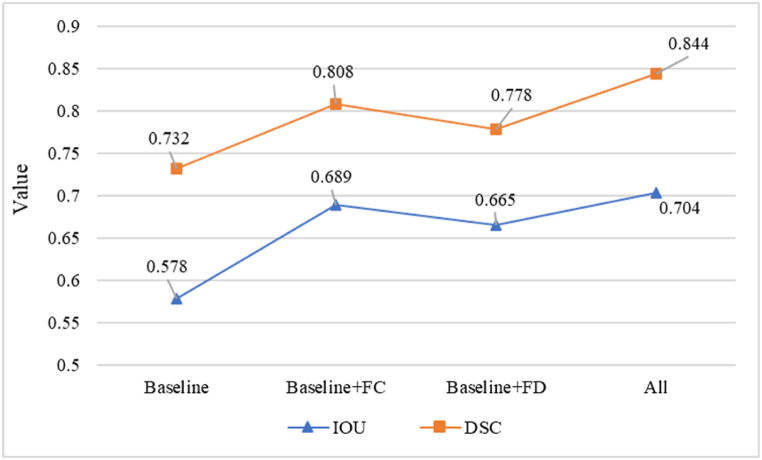
The IOU and DSC scores of baseline with FCA and FDA.

Fig. 15 illustrates how the FCA and FDA modules enhance the segmentation capability of pathological images based on the original model. FCA improves the model's segmentation ability by centrally aligning features in the images, while FDA distributes the alignment of features. We can observe from the images that the generalization ability of the FDA-only model is slightly weaker than that of the FCA-only model. This is because, without the alignment of feature information centers, only distributed alignment may produce inaccurate feature matching between different classes. Likewise, the absence of central alignment weakens the model's capability to generate accurate predictions from new data sampled from the true probability distribution. Therefore, by integrating the FCA and FDA models, the segmentation ability of pathological images is optimized to its fullest extent.

## 5 Conclusion

Based on this, this study proposes a feature alignment-based detail recognition strategy for pathology image segmentation (FASNet). In this study, we use a novel model (UNW) in generalized segmentation in the semantic perception domain to optimize the precision, accuracy and generalization ability of image segmentation in some complex tasks. The UNW network provides more explicit information about the target category by segmenting the image. The experimental evaluation metrics show that the model we used requires only a small amount of computational resources to achieve high segmentation accuracy and precision in a short period of time. In addition. Ablation experiments confirm the contribution of the module we employ to improve the efficiency of the model. Using this approach to process pathology images to provide medical diagnostic information can help specialists diagnose and treat patients faster and more accurately.

The current model still has room for improvement in terms of accuracy and robustness. In future research, we intend to integrate the feature alignment modules FCA and FDA into diverse backbone networks and enhance instance normalization and instance whitening to further enhance the model's accuracy and robustness in unknown target domains. Additionally, we aim to combine different pathology images to facilitate the diagnosis of multiple diseases.

## CRediT authorship contribution statement

**Keke He:** Writing – original draft. **Jun Zhu:** Writing – original draft. **Limiao Li:** Writing – review & editing. **Fangfang Gou:** Writing – review & editing. **Jia Wu:** Writing – review & editing.

## Ethical and informed consent for data used

Not applicable.

## Data availability

Data used to support the findings of this study are currently under embargo while the research findings are commercialized. Requests for data, 12 months after publication of this article, will be considered by the corresponding author.

## Funding

This work was supported in the general project of Changsha Technology Bureau (Grant No. KC1705026) and 10.13039/501100004735Natural Science Foundation of Hunan Province (Grant No.2020JJ4647, Grant No.2020JJ6064), and the 10.13039/501100004735Hunan Provincial Natural Science Foundation of China under Grant 2023JJ30701 and Grant 2023JJ60116.

## Declaration of competing interest

The authors declare that they have no known competing financial interests or personal relationships that could have appeared to influence the work reported in this paper.
